# The Technique of the Extraction of Teeth, Including the Immediate Difficulties and Complications Arising Therefrom*Prize-winning Thesis for the Newland-Pedley Gold Medal for Operative Dental Surgery at Guy’s Hospital, London.

**Published:** 1923-10

**Authors:** Martin Braun

**Affiliations:** England


### THE TECHNIQUE OF THE EXTRACTION OF TEETH, INCLUDING THE IMMEDIATE DIFFICULTIES AND COMPLICATIONS ARISING THEREFROM *

BY MARTIN BRAUN, B. A., L. D. S., ENGLAND

### I. Introductory

The operation of tooth extraction is at the present day correctly regarded as a surgical operation, which accordingly demands all the skill, care and forethought, precision of methods, and refinement of instruments which are associated with surgery as an art and a science. To-day, as

* Prize-winning Thesis for the Newland-Pedley Gold Medal for Operative Dental Surgery at Guy's Hospital, London.

was the case in preanæsthesia times, it must still be regarded as desirable to perform this operation with speed whether, as in the case of nitrous oxide anæsthesia, the reason be the short time the anæsthesia lasts, or whether, as in local anæsthesia, it be necessary to cut as short as possible the period of shock to and strain upon the nervous system, but speed at the expense of skill and science in performing the operation can not be excused. Up to a certain point, then, it is correct to say of tooth extraction that "any operation is quickly enough done that is well done."

From the many cases seen from time to time where teeth have been extracted without due regard to the fact that the operation is a surgical one and the operator dealing with living vital tissues which can be injured in a variety of ways, and the probability of shock to the human system in forcibly removing an organ so firmly implanted as a tooth, one must conclude that the operator must possess not only wide theoretical knowledge as to methods for all cases that may arise, instruments necessary, precautions to be taken before, during and after the operation, but must also possess a high degree of technical ability and practice.

### II. Care and Ppeparation of Patient

In dealing with his patients, the operator should exercise his powers of observation in each case as to the particular method to be pursued; in the first place as to which method of anæsthesia will be advisable (thus, wherever possible, only the more strong-minded, calm type should be selected for local anæsthesia, and nervous and hysterical subjects be persuaded to have a general anæsthesic, all other things being equal). Much of the success of a really scientific and surgical performance of extractions lies in judging the patient's mentality correctly and treating him or her accordingly. The operator should in all that he does, alike before, during and after the operation, remain calm and unexcited, and by his manner inspire confidence in his patient, a confidence which *can* be obtained with a little trouble, and once this point is reached it is possible to do almost anything one wishes, and most patients will then even submit to quite a fair amount of unavoidable pain with little complaint. The operator should be kind, considerate and gentle both in what he says and in his handling of the patient; sometimes it is an advantage to explain in simple language what one proposes doing: at other times this would be a great mistake, according to the subject with whom one is dealing. Fischer says that an unqualified promise of painlessness should on no account be given, rather should the operator explain that there may be some pain and thus prevent suspicion, while at the same time by endeavoring to operate as rapidly and painlessly as possible, pain is often reduced to such a minimum that the patient's entire confidence is gained.

There should further be no unnecessary display of instruments or other suggestions likely to be unpleasant to the patient.

### III. Instruments Used in Extraction

The instruments used for extracting teeth are nowadays, apart from props and gags, of two kinds:

1. Forceps;
2. Elevators.

1. Extraction forceps are made as a rule of nickle-plated steel in two pieces, and consist of two blades and a pair of handles, which may be either curved or straight; in some forceps one handle has a special bend in it to allow of a safe grip and therefore to prevent the hand from slipping up along the handles which are serrated to give a better grip. The forceps are to form part of a long lever, of which forceps, hand and arm all form part, and this lever is to act upon the tooth once this is firmly gripped between the blades. It is essential that the greatest possible mechanical advantage should be obtained from the instrument used, so that the ratio, distance from pivot of operating blades to end of handles: distance from pivot to end of the blades, should be as large as possible, thus the blades should not be too long, but just sufficient to allow practically the *whole* tooth to stand vertically between them, and the handles should be comparatively long —the above-mentioned ratio should be at least about 5:1. In some forceps, used for special cases, a certain amount of mechanical advantage is lost, as in the special type of bayonet forceps used for upper wisdom teeth, where the double elbow which attaches the blades to the handles is very long. In this instrument the handles should be made correspondingly larger. The most efficient (mechanically) forceps are upper "straights" where the forceps acts in the same straight line as the body to be removed. In upper bayonets there is considerable loss of mechanical advantage though this is opposed by the fact of the shape being essential for certain teeth such as upper second bicuspids and molars.

The blades of the forceps should be grooved on their inner aspect and should taper off to a sharp edge and a rounded end and the blades should not enclose a large space between them so as to give greater driving power to separate alveolus from root.

For the maxillary incisors, canines and often first bicuspids "straights" are used, a heavier type for canines which are as a rule more firmly implanted and usually have larger roots than incisors.

For maxillary second biscuspids, broken down molars or molars roots, the "bayonet" type of forceps is employed, many operators using these also, usually of a heavier pattern, for complete upper molars in preference to "full" uppers. The "bayonets" have blades similar to the "straights," but not in the same straight line with the handles (much like a bayonet), so as to allow the hand and handles to clear the lower teeth. A special type of "bayonet" in which the blades are not parallel to the handles is used for teeth which are inclined backwards, thus still allowing the blades to be applied along the long axis of the roots and yet allowing of clearing the lower teeth. Where "bayonets" are not used for complete upper molars, "full" uppers are employed, one for each side of the mouth. These have one ordinary blade for the palatine root and a blade which is divided up by a small projection into two parts, so that not the buccal *roots* but the portion of the tooth between the roots is grasped buccally. This blade can not be driven up the root, and therefore gives a tendency to fracture of the tooth.

Another type of forceps used by some operators for upper premolars and molars is the Read's forceps, where the blades are curved. These are useful sometimes, especially for removing roots, but the disadvantage is that the blades being curved do not rest along the entire length of the root but cross it to a certain extent, and are therefore liable to slip off and to destroy some of the mechanical advantage.

Lower forceps are of two kinds: "hawksbill" roots with blades similar to upper "straights," except placed at right angles to the handles, and "full lowers," which have both blades divided up as in the buccal blade of "full" uppers so that here the roots are not grasped by the blades at all. It is usual to have two or three pairs of "hawksbill," ranging from those with heavy blades for large teeth, medium for medium-sized teeth or broken-down teeth, and fine for small teeth, roots, or where the teeth are much crowded.

Besides the above there are other forms of forceps with blades of unequal size or width, both in uppers and lowers, for displaced teeth in which the ordinary size of blade could not be inserted; blades may also be of unequal length. There are further both upper and lower "root-splitting" forceps for separating the roots of a badly broken-down tooth which can not for some reason be removed in one, but of which the roots, when separated, can easily be removed one at a time. For children's teeth it is usual to use somewhat smaller forceps than the ordinary, especially in lowers where the mouth often can not be opened sufficiently wide to enable the forceps to be applied to the tooth vertically if the blades are too long and hence strike the upper teeth.

2. Elevators are of two kinds, straight and curved, and though theoretically they can be used to remove any tooth, they are used almost exclusively for mandibular third molars, sometimes also for mandibular first and second molars or molar roots. They are made nowadays in one piece with a large tapering handle with square edges which can be firmly grasped in the palm of the hand. The blade is in each case in the same long axis as the handle, the straight being rounded on one side and flat and serrated on the other near the end, which is gently rounded off and sharp so as to cut into the cementum of the root. Curved elevators are similar to the straight, but the blade is bent to form a right angle about one centimeter from the end, and this end is somewhat concave on the side which is applied to the tooth to be extracted. The straight elevator may be used on the left or on the right side; the curved is made in pairs, one for each side of the mouth.

In using general anæsthetics it is necessary to keep the mouth open by mechanical means, and for this purpose either metal props are used, consisting of two serrated somewhat concave flanges united by a short rod, the prop being inserted between upper and lower teeth of one side, or Mason's or similar gag, which has a scissor-like action, and can be opened to a small or large degree as required.

### IV. Position of Chair and Head-Rest, of Patient and Operator, and Use of Left Hand

### Examination of Patient

The patient should be seated almost upright in a comfortable pump chair, preferably one with no bracket-arm attachment, the head should not be bent back but should be practically in a line with the trunk, and the head-rest adjusted accordingly, and moreover in such a way that it supports both neck and head. In extracting maxillary teeth the chair should be pumped upwards so that the operator standing in front and a little to one side of the patient, as will be described presently, does not need to bend down in a cramped attitude in order to see the tooth to be removed. This will enable him also to work with the arm almost fully extended and give him more power for driving upwards than if the arm were flexed. For the mandibular teeth the chair should be let down as low as possible; it is sometimes even necessary for the operator to stand on a low stool so that he can exert a maximum of force downwards in driving home the blades, and at the same time see the field of operation without difficulty. The position and use of the left hand are of very great importance. For the mandibular teeth on the right side the operator should stand behind the patient and a little to the right side so as to lean over the patient's right shoulder; the left arm should encircle the patient's head and grasp the alveolus with the index finger on the buccal side and the thumb on the lingual. These fingers serve not only to guide the blades into the correct position in driving them home, but also by grasping the alveolus firmly, protect it form fracture, and at the same time protect the tongue and cheek from harm. They also (especially the thumb) assist by pressing from the top in forcing the blades home, and serve to compass the alveolus after the tooth is removed, thus preventing excessive after-pain and hemorrhage. The remaining fingers of the left hand support the chin and thus keep the head up. By using the left hand in this way, complete control over the patient's head and mouth is retained, and the head can be moved round into the best position. For the mandibular teeth of the left side the operator usually stands in front of the patient and on his right, leaning across the patient.

In this case the left hand is inserted so that the index finger again lies buccally, and the second finger lingually with the alveolus between them acting again as above, and the thumb supports the patient's chin. Occasionally in difficult cases, a finger of the left hand may be required to press on the top of forceps as mentioned above. For the left mandibular teeth, William Guy of Edinburgh (see Norman Bennett's "Science and Practice of Dental Surgery") prefers to stand on the patient's left. He says:

"The left knee should be slightly bent, the side of his left thigh pressing against the arm of the chair . . . his trunk inclined a little backwards and to the left. The alveolus, as always, is firmly grasped between the finger and thumb of the left hand with the other fingers under the jaw."

The anterior mandibular teeth can be removed when the operator is behind the patient, as described above, and when he is in position for removing the left mandibular teeth. For the maxillary teeth the operator stands on the patient's right and in front, with his thighs against the arm of the chair, and his body poised in such a way as to give a good view of the upper teeth, and yet not interfere with his freedom of action. As before, the alveolus on both sides of the tooth in question must be firmly grasped between forefinger and thumb of left hand, with the same advantage, as explained above; on the right side the thumb keeps away the cheek on the left side, and mostly, for all the incisors, the forefinger keeps the lip away. The importance of always having the fingers of the left hand in the correct position, and doing their work until the tooth is out of the mouth must always be realized. Once these actions of the fingers of the left hand have been acquired, they become almost mechanical.

### V. Asepsis—Arrangement of Instruments and General Precautions

Before commencing the operation, the question of asepsis should be very carefully considered, and the general rule may be laid down that anything which is to come into contact with the mouth should be carefully boiled and rendered sterile, thus forceps, elevators and gags, probes, mirrors and sponges should be treated in this way, and then kept in dishes of Lysol, or other suitable antiseptic, and napkins, cotton wool, etc., should be sterilized by dry heat. No risk, however small, of introducing bacterial infection from without, should be taken, especially as in extraction of teeth the tissues are lacerated to some extent and therefore present a ready mode of entrance for bacteria into the bloodstream. A mouth which is in a septic condition and with deposits of tartar on the teeth should be first subjected to careful scaling and cleansing, and a suitable mouthwash (*e. g.*, one containing carbolic acid) be prescribed to be used frequently before the operation. It is, moreover, a wise precaution to sterilize the field of operation by rubbing the tissues surrounding the tooth or teeth to be extracted with tincture of iodine. In the case of local anæsthesia, this is, of course, absolutely necessary.

Further, the operator's hands and wrists should be carefully scrubbed with soap and hot water and a nail-brush, and immersed in an antiseptic, such as Lysol or 1 in 1,000 mercuric chloride solution, and he should not touch anything but forceps (with right hand only) and patient's mouth (with left hand only) until the operation is over. For very septic or for syphilitic cases, the operator should wear india-rubber operating gloves. The instruments should be arranged in a handy position in some kind of order, so that any which may be required during the operation may be readily found without loss of time; they should lie in shallow dishes containing suitable antiseptics and forceps should be separated out into uppers and lowers. Everything which might possibly unexpectedly be required should be accessible, such as napkins, sponges, hot water, stimulants (such as aromatic spirits of ammonia), etc., etc. In deciding on extraction, the patient's mouth should be systematically examined with mirror and probe in a certain definite order and points of interest and importance noted.

### VI. Actual Procedure—Anatomy of Parts—Instruments Used in Each Case and Method of Grasping

In proceeding to the actual extraction of the tooth or teeth in question, the patient having been placed in the correct position and the operator having taken his place, if forceps are to be used these should be firmly grasped in the right hand with the handles crossing the palm and the thumb lying along the handles and somewhat between them. The handles should be grasped as near their extremities as possible in order to have the maximum of power, and the little finger should rest between the handles and on the inner (smooth) surface of the lower so as to have a ready means of opening the forceps, if necessary, and retaining greater control, therefore, over the instrument. This finger will also help to prevent the hand from slipping up towards the blades with consequent almost complete loss of power. The alveolus having been grasped in the correct way with the left hand, the blades are now applied to the tooth, usually the lingual blade first, and then the labial, and by steady pressure driving up along the root to a sufficient depth, thus driving a wedge between root and alveolus, and the root is then firmly held. A tooth has not to be ''pulled'' out, but it is necessary rather to bend the alveolar plates so as to make room for the roots to pass out, at the same time loosening the root from its attachment which is the periodontal membrane, and then simply to bend the tooth outwards and remove it from the mouth.

1. *Maxillary Teeth.*—Straight forceps used for maxillary teeth. The roots of the incisors are cylindrical in shape so that if a rotatory motion be given to the root it is soon loosened and can be removed. In difficult cases a little bending may also be necessary, in which case it is bent outwards a little and rotated. For canines, a somewhat heavier pair of forceps is usually required and the movement used here should be rather an outward and slight inward one, and very little rotation, as the canine root is somewhat triangular in cross-section. Upper first premolars can usually be removed with straight forceps also and here, as the roots are usually bifid, the tooth is moved outwards then inwards and so on until it is sufficiently loose to allow of its being bent outwards completely and removed. Second premolars are usually removed with "bayonets," sometimes with Read's forceps, and here again the alveolus is bent first outwards, then a little inwards, again outwards and then removed. For upper first and second molars the forceps are selected as explained previously. Having obtained a grip of the tooth, pressure is exerted outwards first and then inwards, and these movements repeated until the tooth is sufficiently loosened to admit of its complete removal outwards. Upper third molars are extracted in much the same way, except that the forceps usually require a greater curvature, as these teeth are usually somewhat inaccessible, and the bending is more inwards than outwards (see below). When "bayonet" forceps are used for removing upper molars (these teeth usually have one palatine and two buccal roots), the inner blade should first be applied to the large palatine root, then the outer blade to the antero-buccal root, if possible, but if badly decayed, to the postero-buccal and the movements carried out as above.

It is necessary to remember the positions in which these roots are normally found so that the blades may be accurately affixed. As these roots are usually slightly curved in a postero-buccal direction, the forceps should be given a slight twist towards the front of the mouth as the tooth is being removed.

In considering the alveolar border of the maxilla, it must be remembered that the outer alveolar plate is much thinner than the inner, and it is for this reason that the bending is always first done in an outward direction, except in the third molar region where the bone is considerably thickened and the movement is, therefore, mostly inwards. Roots when separated from one another are usually readily removed, once grasped, by a rotatory movement of the forceps.

[N. B.—An important point to bear in mind in extracting *upper* teeth is that while bending outwards and inwards the driving upwards should be still to some extent continued, as this will prevent the blades from slipping off, especially in very firmly implanted teeth.]

2. In the mandible again the outer plate of the alveolar border is the thinner. The anterior teeth are firmly grasped as far down as possible by driving the blades downwards between root and alveolus with a steady pressure, then compressing the blades, and as all these teeth are somewhat flattened from side to side, they are not rotated but moved first upwards, then inwards, and these movements repeated until the tooth is sufficiently loosened to be removed in an outward direction. The premolars have cylindrical roots and are therefore rotated, and, if necessary, also bent outwards and then inwards and removed in an outward direction. Care must be exercised with these teeth as they are liable to leave the socket suddenly, and may result in injury to the upper teeth by a blow from the forceps. For lower first molars, either full lower or ''hawksbill'' root forceps may be used; if the former, the blades must be applied so as to grip the tooth as far down as possible linguo-buccally, with the point of each blade in the space between the anterior and posterior roots. Force is then applied in an outward and inward direction until the tooth can be taken outwards. In the region of the second lower molar, and much more so in that of the third, the bone is considerably thickened buccally, so that while they may be treated similarly to first molars the bending movement should be first inwards and only very little outwards, and so on, otherwise there is danger both of fracture of the roots and of the outer alveolar plate and also of production of a wide socket, which will be painful from the stretching of the gum over the bone, and slow healing will result. If "hawksbill" root forceps are used instead of full lowers, the blades are usually applied to the anterior roots, but in cases where the roots are very carious the blades are applied to the least carious. Care should be taken to apply the blades in the long axis of the tooth and to apply the force gradually without jerky movements and at right angles to the tooth. As lower molar roots, especially second and third, tend to curve somewhat backwards, the hand should be given a twist backwards and upwards in removing these teeth so as to disengage more easily the tooth from the socket.

Lower third molars are very often removed with forceps as explained above, but the curvature of the roots of these teeth renders them peculiarly liable to fracture, and it is advisable to use an elevator instead. The straight or curved elevator may be used, though the straight is considered a somewhat dangerous instrument and must be handled with great care and always supported. In using the straight elevator, the handle is grasped firmly in the palm of the hand with the forefinger lying along the blade and resting on the flat side quite near the end of the blade. It is the flat side which faces the tooth to be extracted and the sharp end is driven down between this tooth and the one anterior to it, being inserted from the side and forced well down until in contact with the root, when the handle is depressed and slightly twisted in a direction opposite to that in which the tooth is to move. The blade should cut into the cementum. The fulcrum consists partly of the tooth anterior to the one in question, which should be supported by the thumb of the left hand, and partly by the forefinger lying along the blade of the elevator, or if there is no mesial tooth the alveolus, gum and thumb of the left hand are used as a fulcrum. The tooth will then gradually be tilted backwards, describing a curve corresponding to the curve of the roots, and will either be completely ejected or so loosened that it may then be simply lifted out with a pair of forceps.

If one depression of the elevator only slightly loosens the tooth, the blade may be shifted lower down the root and a new point of contact obtained so as to have the maximum mechanical advantage of the lever in use. The action is very much the same on both sides of the mouth, and one straight elevator serves for both sides. If curved elevator be used two (left and right) will be required, and these may be applied to the mesial or distal surface of the tooth as occasion demands. For third molars it is applied to the mesial surface and inserted from the buccal side and pressed well down with the forefinger lying along the blade. It is then given a turning movement and is also somewhat depressed and the tooth is forcibly ejected as before by simple levering. This elevator may be used also for second and first molars, and more particularly for roots of these teeth for which it is often an invaluable instrument. The fulcrum, as before, is the tooth in front or behind as the case may be, or the gum and alveolus and operator's finger or thumb. In using elevators it is even more important that the left hand be used correctly than with forceps, as these instruments can inflict serious wounds if allowed to slip, especially as considerable force is applied in their use. The soft parts should be carefully protected and the instrument correctly held, so that if it should slip it can not move far.

